# Analysis of narrative assessments of internal medicine resident performance: are there differences associated with gender or race and ethnicity?

**DOI:** 10.1186/s12909-023-04970-2

**Published:** 2024-01-17

**Authors:** Robin Klein, Erin D. Snyder, Jennifer Koch, Anna Volerman, Sarah Alba-Nguyen, Katherine A. Julian, Vanessa Thompson, Nneka N. Ufere, Sherri-Ann M. Burnett-Bowie, Anshul Kumar, Bobbie Ann A. White, Yoon Soo Park, Kerri Palamara

**Affiliations:** 1 Department of Medicine, Division of General Internal Medicine, Emory University School of Medicine, 80 Jesse Hill Jr Dr SE, Atlanta, GA 30303 USA; 2 Department of Medicine, Division of General Internal Medicine, University of Alabama Birmingham School of Medicine, Birmingham, AL USA; 3 Department of Medicine, University of Louisville, Louisville, KY USA; 4 Departments of Medicine and Pediatrics, University of Chicago, Chicago, IL USA; 5 Department of Medicine, Division of Hospital Medicine, University of California, San Francisco, CA USA; 6 Department of Medicine, Division of General Internal Medicine, University of California, San Francisco, CA USA; 7 Department of Medicine, Division of Gastroenterology, Massachusetts General Hospital, Boston, MA USA; 8 Department of Medicine, Endocrine Division, Massachusetts General Hospital, Boston, MA USA; 9 Massachusetts General Hospital Institute of Health Professions, Boston, MA USA; 10 Department of Medical Education, University of Illinois Chicago, Chicago, IL USA; 11 Department of Medicine, Massachusetts General Hospital, Boston, MA USA

**Keywords:** Assessment, Medical education, Graduate medical education, Gender, Race, Ethnicity, Bias, Equity

## Abstract

**Background:**

Equitable assessment is critical in competency-based medical education. This study explores differences in key characteristics of qualitative assessments (i.e., narrative comments or assessment feedback) of internal medicine postgraduate resident performance associated with gender and race and ethnicity.

**Methods:**

Analysis of narrative comments included in faculty assessments of resident performance from six internal medicine residency programs was conducted. Content analysis was used to assess two key characteristics of comments- valence (overall positive or negative orientation) and specificity (detailed nature and actionability of comment) – via a blinded, multi-analyst approach. Differences in comment valence and specificity with gender and race and ethnicity were assessed using multilevel regression, controlling for multiple covariates including quantitative competency ratings.

**Results:**

Data included 3,383 evaluations with narrative comments by 597 faculty of 698 residents, including 45% of comments about women residents and 13.2% about residents who identified with race and ethnicities underrepresented in medicine. Most comments were moderately specific and positive. Comments about women residents were more positive (estimate 0.06, p 0.045) but less specific (estimate − 0.07, p 0.002) compared to men. Women residents were more likely to receive non-specific, weakly specific or no comments (adjusted OR 1.29, p 0.012) and less likely to receive highly specific comments (adjusted OR 0.71, p 0.003) or comments with specific examples of things done well or areas for growth (adjusted OR 0.74, p 0.003) than men. Gendered differences in comment specificity and valence were most notable early in training. Comment specificity and valence did not differ with resident race and ethnicity (specificity: estimate 0.03, p 0.32; valence: estimate − 0.05, p 0.26) or faculty gender (specificity: estimate 0.06, p 0.15; valence: estimate 0.02 p 0.54).

**Conclusion:**

There were significant differences in the specificity and valence of qualitative assessments associated with resident gender with women receiving more praising but less specific and actionable comments. This suggests a lost opportunity for well-rounded assessment feedback to the disadvantage of women.

**Supplementary Information:**

The online version contains supplementary material available at 10.1186/s12909-023-04970-2.

## Background

Inequities associated with gender and race and ethnicity threaten the integrity of assessment [1, 2]. Evidence suggests disparities associated with gender and race and ethnicity occur in quantitative and qualitative learner assessments in medical education [2–15].

Most evidence regarding qualitative assessment in medical education has focused on differences associated with gender in the language used and traits ascribed to learners [7–12]. Less clear is whether gender may affect other aspects of narrative comments such as emotional tone or level of detail. Limited evidence suggests there may be gender-based differences in the tone and consistency of this assessment feedback [12, 13].

Exploring potential differences in narrative comments is important as these assessments serve a vital role in competency based medical education [16]. Narrative comments provide context to quantitative ratings, inform decisions about resident progress in training, and also play an important formative role as developmental feedback for learners [17]. Moreso than ratings, comments are sourced for programmatic letters of recommendations for awards like chief resident, employment and fellowship opportunities [18, 19]. Disparities in these qualitative assessments could have negative effects on learner growth and opportunity.

This study aims to explore differences based on gender or race/ethnicity in the characteristics of qualitative assessments of Internal Medicine (IM) residents in the United States.

## Methods

We applied content analysis to explore characteristics of narrative comments included in faculty assessments of IM resident performance [20, 21].

### Data

Data included clinical performance assessments of IM residents during general medicine inpatient rotations from the 2016–2017 academic year at six US IM residency training programs.

In the US, IM resident clinical educational experiences generally occur in blocks of 2–4 weeks, termed clinical rotations, in which residents provide patient care under the supervision of faculty. Faculty assess resident clinical performance in these rotations using the Accreditation Council on Graduate Medical Education (ACGME)’s core competency framework [22]. Typically, clinical performance assessments ask faculty to provide both numerical ratings of resident performance and narrative comments about the residents performance.

These clinical performance assessments communicate information about resident performance to both the trainee and program. Performance assessments play a dual role of informing decisions about learner progress while also providing meaningful feedback to guide learning [17]. This formative role is emphasized as the ACGME requires programs to facilitate resident review of these assessments and use the information to reinforce strengths and modify deficiencies [23].

This study focuses on the written comments provided in clinical performance assessments and does not include verbal feedback to trainees during rotations as that data is not collected routinely. We use terms qualitative assessment (assessments using non-quantitative data), narrative comments (written commentary), and assessment feedback (formative comments included in an assessment) to refer to the textual information provided in response to open-ended questions within these clinical performance assessments [24–26].

Each progarm in our study used assessment tools that asked faculty to quantitatively rate resident performance as well as provide narrative comments about the resident’s performance. Three programs in our study asked about resident strengths and areas for improvement while the remaining three programs queried about overall resident performance and also allowed for open text comments organized by the six ACGME core competencies [22]. Comments were grouped into domains based on question stem: strengths and areas for improvement and overall comments and competency-specific comments.

We also collected data on resident characteristics (race and ethnicity, gender, post-graduate year (PGY), baseline IM In-Training Examination (ITE) percentile rank), faculty characteristics (gender, specialty, academic rank, residency educational role), and rotation setting and date. Gender designations were determined by participants’ professional gender identity (gender identity used in their professional role as residents) as known to the residency program director. We acknowledge that one’s professional gender identity may differ from their gender identity expressed in other settings. Race and ethnicity designation was self-reported on residency applications, and we utilized the Association of American Medical Colleges (AAMC)’s definition of URiM as those who are underrepresented in medicine relative to national and local demographics [27]. Faculty gender was determined from institutional profiles; faculty race and ethnicity information was not collected. In our analysis, cis and trans women were included as women and cis and trans men were included as men. Data was extracted from program management systems by program staff and was de-identified before analysis by removing names and gendered pronouns.

### Qualitative content analysis

We used content analysis to explore two key characteristics of comments: specificity and valence [20, 21]. We employed a multistage, multi-analyst approach that included familiarization and immersion with data, generating a coding frame through iterative coding and discussion, and applying this coding frame with weekly review of coded data and discussion to achieve consensus [28–30]. Research team included men and women as well as physicians and non-physicians. Some team members identified as URiM physicians. The blinded coding team included three physicians (RK, ES, JK) with experience in IM resident assessment and the IM milestone assessment framework [30]. Two investigators independently coded each comment using qualitative coding software (MaxQDA) and reconciled differences via discussion. Cohen’s kappa measure of interrater reliability was > 0.80. We analyzed all comments to strengthen the generalizability of results.

### Characteristics of comments

Informed by prior work, we developed two codes (specificity and valence) to capture key characteristics of an assessment comment [12, 31]. Comments from each evaluation were rated in these dimensions. See Table 1.

**Table 1:** Framework for Comment Characteristics of Specificity and Valence used in study of Association of Gender and Resident Race and Ethnicity and Narrative Comments from Internal Medicine Resident Performance Assessments

Comment Characteristic	Description	Scale	Details
Specificity	Highly Specific	3	References more than 3 core competencies OR includes 2 specific items (includes specific examples of something done well or an area for improvement or action items)
	Moderately Specific	2	References 2 to 3 core competencies OR includes 1 specific item (includes specific examples of something done well or an area for improvement or action item) in a core competency
	Mildly Specific	1	References 1 core competency
	Non- specific	0	References 0 core competencies
Valence	Very Strong Praise	+ 3	Very strongly positive, very high praise
	Strong Praise	+ 2	Strongly positive, high praise
	Praising	+ 1	Fairly positive, moderate praise
	Neutral	0	Neutral
	Critical	-1	Fairly negative tone, moderate criticism, includes 1 ‘red flag’ about performance
	Strong Criticism	-2	Strongly negative tone, critical, includes 2 ‘red flags’ about performance
	Very Strong Criticism	-3	Very strongly negative tone, critical, includes 3 ‘red flags’ about performance

Specificity refers to the level of detail and degree of actionability of the comment. Specificity was rated on a 4-point scale from non-specific to highly specific based on the number of competencies referenced and the inclusion of specific examples of resident performance and action items for improvement.

Valence refers to the overall positive or negative tone or orientation (praising or critical) of the comment. Valence was rated on a 7-point scale based on the tone and language used to reference performance. Importantly, inclusion of areas for growth did not necessarily detract from the praising or positive orientation of the comment and we differentiated between comments framed as developmental feedback and those phrased as “red flags” for serious concern.

### Analysis

We examined the potential relationships of the specificity and valence of narrative comments in an evaluation with resident gender, resident race/ethnicity, resident PGY, and faculty gender using multilevel regression.

We controlled for type of comment (i.e., Overall Performance, Strengths and Areas for Improvement, Competency-specific comments) and quantitative rating of the evaluation, as both may relate to the actionability and tone of narrative comments in an evaluation [31]. We also controlled for the other characteristics of a comment (specificity or valence) as conceptually we suspected that comments that are critical may also be more actionable. To control quantitative ratings, we used a standardized composite competency score for each evaluation by calculating the arithmetic mean of core competency ratings, which was then standardized based on the score distributions at each program.

We then assessed the relationship between specificity and valence of comments and resident gender, PGY, race and ethnicity and faculty gender using mixed-effects regression, accounting for clustering by learner and faculty within programs. We controlled for standardized composite core competency score, type of comment (Overall Performance, Strengths and Areas for Improvement, Competency-specific comments), other characteristic of comment (specificity or valence), program, rotation setting and date, resident characteristics (race/ethnicity, gender, PGY, and baseline IM ITE percentile rank), and faculty characteristics (gender, specialty, academic rank, and educational role). In our analysis, men and non-URiM residents were used as the reference group.

To demonstrate the validity of our coded constructs, we analyzed the relationship between the quantitative ratings provided in an evaluation and the characteristics of comments (mean specificity and valence).

We report patterns and differences in specificity and valence of narrative comments associated with gender, race/ethnicity and PGY. Given this study uses a positivist and pragmatic approach, we quantitized our data using the scales described and report differences in scale units [32, 33]. At times, we report odds ratios to convey the difference in more accessible terms. De-identified quotes are presented to ensure confidentiality of participants and sites.

Institutional Review Boards at each institution deemed the study exempted. Funding sources were not involved in study design, data analysis and interpretation, manuscript preparation, or decision to approve publication of the manuscript.

## Results

Of 3600 evaluations collected, 3,383 (94%) included narrative comments and were included for analysis (Table 2). Data included this included assessment data for 385 men residents (55.2%) and a 313 women residents (44.8%). Of the faculty, 315 (52.8%) were men and 282 (47.2%) were women. We did not identify any openly gender non-binary participants. Data included 447 assessments of URiM residents (13.2%).

**Table 2:** Qualitative Assessment Data from study of Association of Gender and Resident Race and Ethnicity and Narrative Comments from Internal Medicine Resident Performance Assessments

Number of Evaluations with Comments		3383
Comment Type		
	Overall comments	1959 (57.9%)
	Strengths and Areas for Improvement	1335 (39.5%)
	Competency-specific comments	554 (16.4%)
Resident Gender, N (%)		
	Men	1834 (54.2%)
	Women	1549 (45.8%)
Resident URiM designation, N (%)		
	URiM	447 (13.2%)
	Non-URiM	2936 (86.8%)
Post-graduate year, N (%)		
	PGY1	1980 (58.5%)
	PGY2	823 (24.3%)
	PGY3	580 (17.2%)
Faculty gender, N (%)		
	Men	1880 (55.6%)
	Women	1503 (44.4%)
Faculty rank, N (%)		
	Professor	485 (14.3%)
	Associate Professor	678 (20.0%)
	Assistant Professor, Instructor or Other	2220 (65.6%)
Faculty department, N (%)		
	General Medicine	1607 (47.5%)
	Hospital Medicine	1290 (38.1%)
	Subspecialty	486 (14.4%)
Faculty educational role, N (%)		
	Program Director or Associate Program Director	390 (11.5%)
	Chief Resident	279 (8.2%)

Abbreviations: PGY = post-graduate year; URiM = Underrepresented in Medicine; non-URiM = not Underrepresented in Medicine

Most assessments included overall performance comments (1959 evaluations) or strengths and areas for improvement (1335 evaluations). Data included more assessments for PGY1 residents than PGY2 and PGY3 residents.

Overall, residents received a mean of 4.8 evaluations with comments and faculty provided a mean of 5.7 evaluations with comments in the academic year studied. There was no significant difference in the likelihood of receiving an evaluation without comments between women and men residents (OR 1.56, 95% CI 0.96 to 2.52).

### Comment characteristics: specificity and valence

Table 3 includes representative quotes supporting the specificity and valence scales.

**Table 3:** Characteristics of Narrative Comments from study of association of Gender and Resident Race and Ethnicity with Narrative Comments from Internal Medicine Resident Performance Assessments

Level & Description	Illustrative Examples
**Valence**	
**Very Strong Praise** Very strongly positive, very high praise	*“In 23 years of working with residents I would place (First Name) in TOP 5% of all residents I have worked with. They are an exceptional physician and young person.”* Overall Comment, Man PGY2 resident *“Chief Resident material.”* Overall Comment, Man PGY1 resident *“(First Name) is an outstanding intern. I usually expect such excellent performance in May rather than in November. They have an advanced fund of knowledge. They are thorough, meticulous, has great differentials and plans. They are also very professional and compassionate. (First Name) leads family discussions well beyond their training. It was a pleasure to work with them. They are also helpful to their teammates and shows leadership skills. (First Name) would make a great chief. (Areas for improvement) None. Keep going on your path.”* Strength and Areas for Improvement Comment, Woman PGY1 resident *“(First Name) is a phenomenal team leader, incorporating every member of the team into the patient’s care plan as well as any educational opportunities. Differential diagnoses were complete and appropriately prioritized.”* Strength and Areas for Improvement Comment, Man PGY3 resident *“Dr. (Last Name) was an outstanding role model and an excellent team leader. They functioned at the level of an attending and made smart decisions about patient care. They demonstrated their perfect combination of art and science in their care of their patients. they are a compassionate caregiver. I appreciated the opportunity to work with them and to learn from them.”* Strength and Areas for Improvement Comment, Woman PGY3 resident *“Always the utmost professional.”* Competency Specific Comment, Man PGY3 resident *“Dr. (Last Name) is an exemplary intern, hard-working, professional, compassionate, and efficient, in the top 10% of interns I have taught. Their case presentations were particularly notable - organized and efficient with a clear logical progression and transparent clinical thinking. They also showed keen sensitivity, asking a patient if they could disclose medical information to the patient’s interested friend. Overall, a real pleasure to have on our team.”* Overall Comment, Woman PGY1 resident
**Strong Praise** Strongly positive, high praise	*“Excellent resident! They prepared the interns very well for daily rounds and H and P presentations. Very knowledgeable and reliable, they knew the census well and always followed up. They were willing to take on extra responsibilities and kept family informed. I would love to work with them again in the future.”* Overall Comment, Woman PGY2 resident *“(First Name) overall had an extremely good month as an intern on July (rotation). They knew all the patients on the team, not just their own patients. They had a plan for every patient and challenged themself by looking up any answers they did not know. Really they are performing above the level expected. They should continue to work just as hard throughout residency- will be a very, very good resident.”* Overall Comment, Man PGY1 resident *“(First Name) performed in an excellent fashion during our time together. They were thorough, organized, efficient and always level-headed and committed to doing their best. They are very bright, with an above average fund of knowledge. They made excellent progress in adapting to bedside presentations and making their notes and presentations more concise in response to feedback. They had excellent rapport with the rest of the team and the staff. They are off to a great start! Keep working on efficiency and presentations and notes (these are not problems, just areas they can get better given this is just their second month). Work on confidence and seeking more independence as the year progresses (same comment as above). Don’t be too hard on yourself. You did a great job this month.”* Strength and Areas for Improvement, Man PGY1 resident *“Excellent job in patient care.”* Competency Specific Comment, Woman PGY3 resident
**Praise** Fairly positive, mild to moderate praise	*“(First Name) is progressing appropriately. They are working on improving their efficiency and their knowledge base. They are a good team player and helps teach the medical students. They completed medical records in a timely fashion, with appropriate detail.”* Overall Comment, Woman PGY1 resident *“Overall, a very solid resident with good knowledge, judgment and people skills.”* Overall Comment, Man PGY3 resident *“Dr. (Last Name) is performing at the level expected for their first month on inpatient service. They obtain accurate history and physical. Their medical knowledge is at the level expected for their training. They can continue to expand their medical knowledge through reading and clinical experience. They were reliable to follow-up on patient care during the day. Overall, they did well during this rotation.”* Overall Comment, Woman PGY1 resident *“Very good fund of knowledge, strong data gathering and communication skills, strong ability to formulate a treatment plan -- all reasonably advanced for a first month PGY2. Took on the mantle of team leader comfortably and with confidence. Interns went out of their way to tell me that they enjoyed working with them, and that they created the right balance of oversight and autonomy. Also, took time out to praise team members when they did things right, a nice thing to do.”* Strengths and Areas for Improvement Comment 6048 *“(First Name) was a great intern. They have a good fund of knowledge with no deficits. They are also adept at communicating with patients and handling very difficult patients, without compromising appropriate and good care. I enjoyed working with them and they will be a very good resident.”* Strengths and Areas for Improvement, Woman PGY1 resident *“(First Name) did a nice job coordinating care for their patients and employing the assistance of other health care providers.”* Competency Specific Comment, Man PGY2 resident
**Neutral** Neutral, mix praise and criticism	*“Cheerful July Intern who will improve as they gain confidence and clinical knowledge bolstered by intensive study.”* Overall Comment, Man PGY1 resident *“(First Name) is a warm, earnest, and hard-working intern. I very much enjoyed getting to know and work with them. They have a flare for language and is funny. They are kind at the bedside and always willing to take on tasks. They are an eager learner and engages easily. (First Name)’s work-ups and presentations remain somewhat disorganized and their presentations still lack a flow and cohesiveness that would help the listeners understand their thinking process. They took feedback well about these issues. I think with some further directed and focused work in this area, they will catch up quickly.”* Overall Comment, Man PGY1 resident *“Dr. (Last Name) did well for it being only their second month of residency and being on a difficult service. I am certain with time, they will become an excellent independent physician. Dr. Last Name could expand on their fund on knowledge - particularly in regards to recognizing when patients worsen rather than improving. Also on that note, they could work to be proactive rather than reactive in patient care.”* Strength and Areas for Improvement, Man PGY2 resident *“Efficient, pleasant to work with. Would benefit from more exposure to patients as expected at this stage of career development.”* Strength and Areas for Improvement, Man PGY1 resident *“(First Name) was open to feedback which I provided but did not necessarily solicit my feedback. I did not observe them soliciting feedback from the interdisciplinary team with whom they worked, so I cannot comment on that aspect of their commitment to improve through feedback.”* Competency Specific Comment, Man PGY2 resident
**Critical** Negative tone, criticism, may include red flag(s) about performance	*“Can handle straight forward problems; when there are complicating factors sometimes doesn’t use all the data available and concentrates on the data that reinforce their ideas and does not account for conflicting info.”* Competency specific comment, Man PGY2 resident
**Specificity**	
**Non- specific** References 0 core competencies	*“(First Name) is ready to be a resident. As a resident they will have the opportunity to teach more and lead teams, and they are ready for that next step.”* Overall Comment, Woman PGY1 resident *“Only needs continued experience - is doing everything well for their level.”* Strengths and Areas for Improvement, Man PGY1 resident
**Weakly Specific** References 1 core competency	*“Overall (First Name) did a great job collecting information, analyzing information, and coming up and implementing the plan.”* Overall Comment, Woman PGY1 resident *“This is my second time working with (First Name) on the inpatient setting. They are a very strong intern who is transitioning nicely in taking more responsibility for their patients and will be a great senior resident. They are also very organized, efficient, and again takes ownership of their patients.”* Strengths and Areas for Improvement Comment, Woman PGY1 *“(First Name) did a great job as team leader during our week together. They are an incredibly hard worker who clearly cares deeply about their patients. They could always be relied upon to follow through and do what needed to make sure their patients got their best care possible. (First Name) will clearly continue to do well in their field of choice.”* Strengths and Areas for Improvement Comment, Woman PGY3 resident *“Excellent work with multidisciplinary team.”* Competency Specific comment, Woman PGY1 resident
**Moderately Specific** References 2 to 3 core competencies OR includes 1 specific examples of something done well, an area to improve, or action plan for improvement	*“Thinks critically about patients and practices evidence-based medicine. Elicits feedback about decision-making for diagnostic and therapeutic plans.”* Strength and Areas for Improvement Comment, Man PGY1 resident *“Dr. (Last Name) has deep and thorough knowledge of complex medical care. They are also a trustworthy person who relates to team members in an entirely constructive way. They maintain a posture of respect and care for all the people they work with, including their patients.”* Strength and Areas for Improvement, Man PGY3 resident
	*“(First Name) was wonderful to work with on (rotation). They are thoughtful, patient, kind and a great team player. They connect strongly with patients, is committed to seeing their care through and spending time with them at the bedside. As swing one day they spent most of the morning with a very sick patient on the floor, stepping up to do an ABG and interfacing with ICU several times. (First Name) puts their patients first and is always willing to learn.”* Overall Comments, Woman PGY1 resident *“Very capable resident who provided good care to patients and families. They did struggle with phone communication with support staff such as nurses and clerks though in person communication with staff was fine.”* Overall Comment, Woman PGY1 resident *“(First Name) is a strong intern and could easily finish the year with (receiving) minimal constructive feedback. I encouraged them to get around this by being specific about asking for feedback in real time. For example, when staffing an admission, asking “would you recommend I do that differently next time?” or “if I could have done that even better, what would you recommend?”* Overall Comments, Woman PGY1 resident
**Highly Specific** References 4 or more core competencies OR includes 2 specific examples of something done well, an area to improve, or action plan for improvement	*“Dr. (Last Name) is a highly knowledgeable and efficient intern. They effectively prioritize tasks and navigates the (institution) system to provide the best care for their patients. They promptly complete their clinical notes, which are concise yet always include the key details. They are dedicated to patient care, staying late when necessary, and taking time to communicate effectively with their patients. They were diligent in addressing clinical questions that arose regarding their patients. At the bedside, they always maintain their composure and positive regard for the patient, even in difficult situations.”* Overall Comment, Woman PGY1 resident *“(First Name) did a great job coordinating care for complex patients and assuring appropriate transitions between the hospital and other settings (outpatient, transfer to outside hospital, etc.). Their diagnostic and management plans are appropriate and complete. They do a great job paying attention to details and making sure everything is follow-up on appropriate. They developed excellent rapport with patients and their families and was always thoughtful and considerate.”* Strength and Areas for Improvement Comment, Woman PGY1 resident *“Dr. (Last Name) has an excellent fund of knowledge and is able to manage a broad range of clinical issues independently. They identified infectious disease and antibiotic management as a particular area where they need to improve. They have a highly effective approach to learning, systematically addressing clinical questions using point-of-care resources and the medical literature. They went out of their way to teach the medical students, and taught the team about clinical topics almost every day on rounds. Their bedside manner really communicates concern for the patient. They usually sit on the bed, which helps them establish rapport, and allows for long pauses when patients are dealing with strong emotions. Their notes and oral presentations were concise and complete, demonstrating a thorough understanding of the active clinical issues.”* Overall Comments, Woman PGY1 resident

### Specificity of comments

Specificity refers to the detailed nature or actionability of the comment. Most overall and strength and weakness comments were moderately specific (52.3%).

Non-specific comments (11.2%) included those that did not reference skills or attributes included in the ACGME’s core competencies. These comments often referenced barriers or qualifiers to the faculty member’s assessment of the resident’s performance (i.e., “Interaction was too brief to say”), offered no suggestions (i.e., “No suggestions”), or were not attributable to a core competency (i.e., “Great job”).

Weakly specific comments (22.1%) referenced one core competency, as the following quote illustrates.

*“They are able to recognize when people are sick, make quick decisions, all while maintaining a calm demeanor. They have solid plans for their patients, and I really had to change very little with regards to treatment plan. Overall great job.”* Overall Comment, Man PGY2 resident

Moderately specific comments (52.3%), as illustrated by the following quotes, provided either more breadth by referencing 2 or 3 competencies or depth by including specific examples within a competency such as examples of things done well, skills to be improved, or action plans for improvement.

*“(First Name) did an extremely good job on (rotation) month. They managed the team extremely well. They accurately knew all the details of their patients’ care and formulated excellent patient care plans that efficiently provided excellent care. They communicated effectively with patients and their families. They will make a terrific (future role). A very, very good job; I was fortunate to have them as my upper-level resident.”* Overall Comment, Woman PGY3 resident

Highly specific comments (14.4%) were very detailed and thorough, referencing 4 or more core competencies or included multiple specific examples.

*“Dr. (Last Name) exceeded expectations leading a (rotation) team. Their fund of knowledge and clinical judgment are equally impressive. They were able to balance efficiency with education on rounds, finding teaching moments for the interns but also managing time well so that all of the patients were seen, and the team got to noon conference every day. At the bedside with patients and families they set a great example for the interns, quickly establishing rapport and putting people at ease. They worked extremely well with the nurses, case managers, and other floor staff, who universally praised them. The interns on the team admired and respected them. They set a very high bar for themselves and inspired the rest of the team to do the same. They were reflective about their work, looked for ways to improve, and asked proactively for feedback and suggestions. They are a very effective communicator and was able to galvanize the entire team around a common goal. Dr. (Last Name) is a natural and effective leader and I anticipate will continue to be a leader in the program.”* Overall Comment, Woman PGY2 resident

### Comment specificity and gender, pgy, and race and ethnicity

Controlling for covariates including standardized composite competency rating, comment type, and valence of comment, there was a significant difference in the specificity of comments with resident gender, with women receiving less specific comments than men residents (estimate − 0.07, p 0.002) (Table 4).

**Table 4:** Association of Comment Characteristics with Gender and Resident Race and Ethnicity from study of Narrative Comments from Internal Medicine Resident Performance Assessments

	Specificity of Comments			Valence of Comments		
	Estimate†	Standard Error	*P* value	Estimate†	Standard Error	*P* value
Resident Gender*	-0.07	0.02	0.002	0.06	0.03	0.045
Faculty Gender**	0.06	0.04	0.149	0.02	0.04	0.540
Resident Race and Ethnicity***	0.03	0.03	0.315	-0.05	0.04	0.262

* women residents compared to men residents

** women faculty compared to men faculty

*** URiM residents compared to non-URiM residents

† Based on multilevel regression controlling for standardized composite competency rating, type of comment, comment characteristic (valence or specificity), program, resident characteristics (PGY, gender, race/ethnicity, and baseline In-Training Examination percentile rank), faculty characteristics (gender, department, and education role), rotation time of year and hospital setting where comments are partially cross classified by resident and faculty

Women residents were more likely to receive either no comments or nonspecific/weakly specific comments (adjusted OR 1.29, 95% CI 1.06 to 1.57, p 0.012). Women residents were less likely to receive very highly specific comments (adjusted OR 0.71, 95% CI 0.56 to 0.89, p 0.003) or comments with specific examples of things done well, areas for improvement, or detailed action items for improvement (adjusted OR 0.74, 95% CI 0.61 to 0.90, p 0.003) than men residents.

**Fig. 1:**
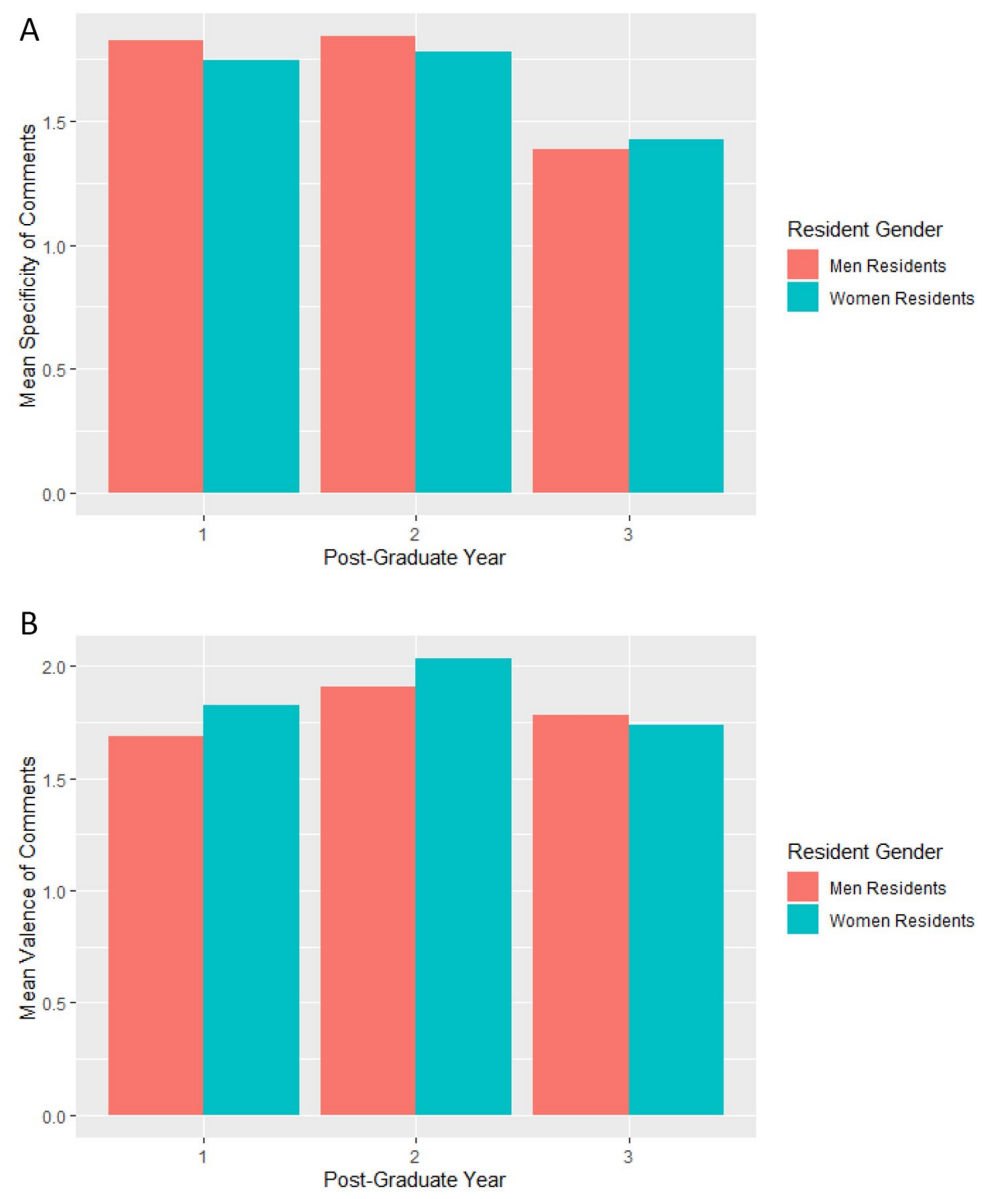
Specificity and Valence of Narrative Comments by Resident Gender and Post-Graduate Year from study of Association of Gender and Resident Race and Ethnicity and Narrative Comments from Internal Medicine Resident Performance Assessments. Panel 1**A**: Mean Specificity of Narrative Comments by Resident Post Graduate Year and Gender. Panel 1**B**: Mean Valence of Narrative Comments by Resident Post Graduate Year and Gender

Overall, PGY1 and PGY2 residents received more specific comments as compared to PGY3 residents (Fig. 1A). The difference in specificity of comments received by men and women residents was most notable and significant in PGY1. In PGY1, the difference in the specificity of comments of men and women residents was significant, with women interns receiving less specific comments than men interns (estimate − 0.11, p < 0.001). See Appendix Table.

There was no significant difference in comment specificity based on faculty gender (estimate 0.06, p 0.15) or resident race and ethnicity (estimate 0.03, p 0.32).

### Valence of comments

Valence refers to the emotional tone (positive or negative) and orientation (i.e., praise or criticism) of comments. Overall, the valence of comments was positive, with most comments providing praise (36.4%) or strong praise (33.7%).

Praising comments (36.4%) described performance as ‘solid,’ ‘effective,’ or ‘very good’ and often noted that performance was at expected level or comparable to peers. The following quote illustrates a mildly positive, praising comment.

*“(First Name) takes great care of their patients. They are very good at data collecting and is doing well this year.”* Overall Comment, Man PGY1 resident

Strongly praising or positive comments (33.7%) often included descriptors like ‘excellent’ and cited performance or skills as advanced or above expectations.

*“(First Name) did an excellent job! They operate at the level of a PGY-3. They did an excellent job identifying and managing some particularly sick patients and I knew I could completely trust their judgment. I encourage (First Name) to continue to work on discharge planning, particularly determining when a patient is appropriate for discharge.”* Strength and Areas for Improvement Comment, Woman PGY2 resident

Very strongly praising comments (25.2%) described performance as ‘outstanding’ or ‘exemplary’ and often noted that the performance stood out from others, was worthy of honor or reward, or ranked highly in the experience of the faculty member.

*“(First Name) is one of the strongest residents with whom I have worked in (number) years. They have all the qualities necessary to be a leader in medicine -- knowledge, skill, kindness, and diligence. (First Name) performed at the highest level in all domains. They would be an excellent chief resident.”* Overall Comment, Woman PGY1 resident

### Comment valence and gender, pgy, and race and ethnicity

Controlling for covariates including standardized composite competency rating, comment type, and specificity of comment, there was a significant difference in comment valence with women residents receiving more positive, praising comments than men residents (estimate 0.06, p 0.045) (Table 4).

Overall, PGY2 residents received more praising comments (Fig. 1B). The difference in valence of comments received by men and women residents was most notable earlier in training. In PGY1, the difference in comment valence for men and women residents was significant, with women interns receiving more positive comments than men (estimate 0.10, p 0.015) (Appendix Table).

There was no difference in valence of comments based on faculty gender (estimate 0.02 p 0.54) or between URiM and non-URiM residents (estimate-0.05, p 0.26).

### Ratings and comment valence and specificity

Standardized composite core competency score was associated with comment specificity (estimate − 0.08, p < 0.001) and valence (estimate 0.46, p < 0.001) such that evaluations with lower ratings included more detailed comments and as quantitative ratings increased, the comments included in that evaluation became more positive (Appendix Figure). There was no significant relationship between specificity of a comment and its valence (estimate 0.02, p 0.147). A comment may be highly positive or praising but not necessarily specific, detailed, or actionable.

## Discussion

In this multisite study, there were notable differences in the characteristics of narrative comments in performance assessments received by men and women residents. Comments about women residents were more positive but less specific and detailed than those of men residents, even when controlling for numerical ratings. These findings are in contrast with a smaller study in a single U.S. anesthesia program which showed no difference in the likelihood of receiving vague feedback with resident gender [34]. However, our findings are consistent with research looking at performance reviews outside of academia, which found women were less likely to receive specific feedback tied to outcomes, and this occurred with both praise and critical feedback [35–37].

We found women received more positively toned comments than men residents while controlling for several variables including the detailed nature of comments and the quantitative ratings accompanying comments. Prior evidence looking at the effects of resident gender on tone of qualitative assessments is limited. A qualitative study of narrative comments in emergency medicine resident assessments noted women residents received more discordant comments, suggesting a mix of praise and criticism across faculty members [13]. Studies of narrative comments in surgical resident assessments have mixed results in terms of gender-based differences in tone of comments [12, 38].

Overall, the differences in specificity and valence of comments received by men and women trainees were most notable earlier in training. For both specificity and valence, the overall differences across training were driven by differences in PGY1. This may be due to the number of evaluations for interns compared to later years. Overall trends in specificity and valence across training years warrant further study.

We found no difference in comment specificity or valence based on gender of faculty assessor. This contrasts with a study of In-Training Evaluation Reports of surgical residents that found women raters provided more positively toned comments than men faculty and comments by women faculty were longer and more detailed than men raters [38].

We found no difference in the characteristics of qualitative comments with resident race and ethnicity. While evidence looking at differences in assessments associated with race and ethnicity is limited, prior work using this same cohort has reported disparities in quantitative ratings with race and ethnicity [5]. The ability to detect differences in specificity and valence of comments related to race and ethnicity may have been limited by low numbers of URiM learners. This may reflect an inability to detect a difference rather than a lack of difference. 

Importantly, there are potential implications for these findings for learners and programs in graduate medical education. Performance assessments play a dual role of informing decisions about learner progress while also providing meaningful feedback to guide learning [17]. Feedback is defined as information provided to a learner regarding aspects of one’s performance or understanding for the purposes of improvement [39, 40]. Considered within a formative framework, narrative comments serve as feedback to learners about their performance to enable their growth and development [26]. Specific and actionable assessment feedback helps acknowledge learner strengths, name areas for development, and provides clearly defined, actionable items for growth.

Qualitative assessments may also influence program leaders’ perceptions of residents. Assessment feedback is often sourced for programmatic letters of recommendations for awards like chief resident, employment, and fellowship opportunities [18, 19]. As such, disparities in assessment feedback may impact resident growth and opportunity.

Receiving weakly specific comments on performance can be seen as a lost opportunity and hinder the overall growth and development of women residents. This is especially concerning given the greatest difference in specificity of comments found earlier in training when residents are in the most formative stage. Taken together, the findings of positive but less specific comments provided to women residents raises the question of whether the comments contained verbiage which could be construed as ‘empty platitudes’ or praise for skills and attributes outside of the core competencies. Further study is warranted to explore gender-based differences in the content of narrative comments.

Importantly, this study only explored the written comments provided in clinical performance assessments and did not include verbal feedback to trainees during rotations. It is possible that the disparities in the specificity and valence seen in assessment feedback may be mitigated by the verbal feedback provided throughout the rotation. In other words, women may receive positive but less specific narrative comments but more actional verbal feedback throughout the rotation. Study is needed to explore gender differences in verbal feedback including willingness to provide and receptivity to feedback.

While the differences in specificity and valence found were small, evidence suggests that even small differences in performance assessments can have a cascade effect and lead to greater disparities in subsequent outcomes [41]. Differences in assessment imply a difference in the training experience of residents and any evidence of disparities should be sufficient to warrant our concern.

Finally, the findings of this study offer a potential focus for interventions to address inequities in assessment. Providing detailed feedback within and across the core competencies that includes specific examples and plans for improvement can be a target of faculty development. Importantly, as this study demonstrates, the detailed, specific nature of narrative comments can be measured and thus monitored as an indicator of assessment quality and equity [31].

Limitations of this work include retrospective, cross-sectional data which does not allow for assessing differences within residents over time. Assessment tools varied across sites, however we used a rigorous approach to enable comparison. Limitations of our data mean we were not able to explore the comments of those identifying as gender non-binary. This study does not account for all the socioeconomic factors that may influence assessment. The study sample is limited to academic institutions in the United States from 2016 to 17 academic year. It may be useful to study a broader sample of narrative comments to see if these differences persist as context changes.

## Conclusions

Our findings suggest there are differences in the characteristics of narrative comments included in performance assessments of men and women trainees, with women receiving more positive but less specific feedback than men. This suggests that disparities in assessments are not confined to ratings or traits ascribed to learners; rather, they manifest in complex ways that can hinder the overall growth and development of women residents. The specificity and tone of narrative comments may be an important target of efforts to promote high-quality, equitable assessment of residents.

### Electronic supplementary material

Below is the link to the electronic supplementary material.

Supplementary Material 1

Supplementary Material 2

## Data Availability

Datasets generated and/or analysed during the current study are not publicly available due to restrictions on sharing assessment data. For further inquiry regarding the study, contact the corresponding author. iv.
