# Supplementary material for: Analysis of narrative assessments of internal medicine resident performance: are there differences associated with gender or race and ethnicity?
Source: BMC Med Educ. 2024 Jan 17;24:72. doi: 10.1186/s12909-023-04970-2 (PMC10795394; doi:10.1186/s12909-023-04970-2)

Appendix Figure 1: Relationships between Comment Specificity, Comment Valence, and Quantitative Ratings from study of association of Gender and Resident Race and Ethnicity with Narrative Comments from Internal Medicine Resident Performance Assessments

Panel A: Mean Specificity of Narrative Comments and Standardized Composite Core Competency Score


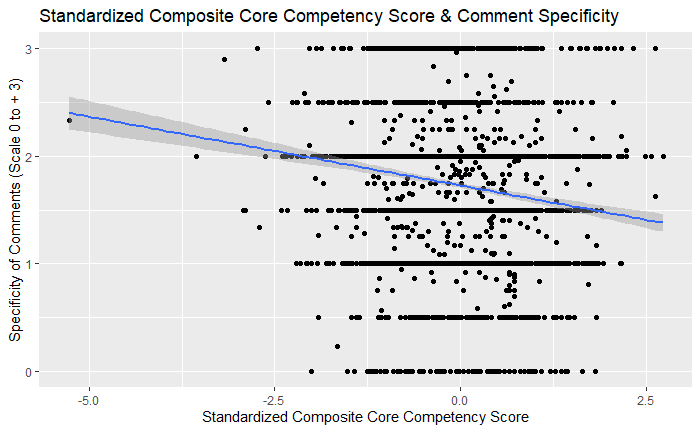


Panel B: Mean Valence of Narrative Comments and Standardized Composite Core Competency Score


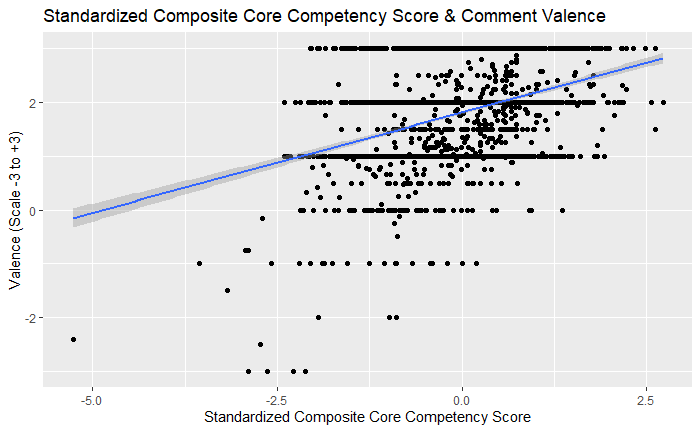


Panel C: Mean Specificity and Mean Valence of Narrative Comments


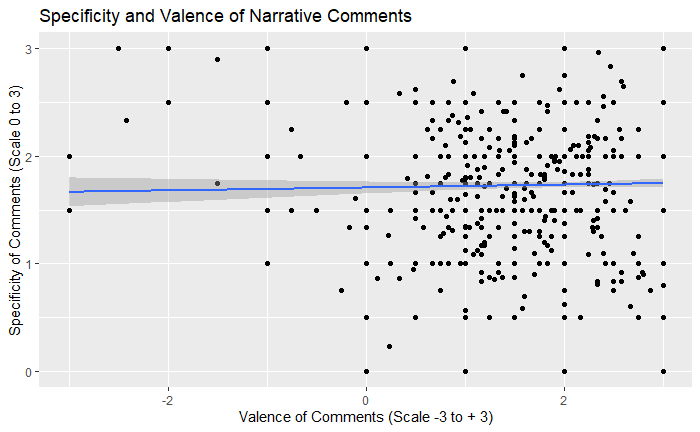

Supplement: Supplementary file 1 — Supplementary Material 1 [file 12909_2023_4970_MOESM1_ESM.docx]
