# Supplementary material for: Analysis of narrative assessments of internal medicine resident performance: are there differences associated with gender or race and ethnicity?
Source: BMC Med Educ. 2024 Jan 17;24:72. doi: 10.1186/s12909-023-04970-2 (PMC10795394; doi:10.1186/s12909-023-04970-2)
Supplement: Supplementary file 2 — Supplementary Material 2 [file 12909_2023_4970_MOESM2_ESM.docx]

Appendix Table 1: Associations of Comment Characteristics with Resident Gender and Post Graduate Year from study of association of Gender and Resident Race and Ethnicity with Narrative Comments from Internal Medicine Resident Performance Assessments

| Appendix Table 1: Associations of Comment Characteristics with Resident Gender and Post Graduate Year from study of association of Gender and Resident Race and Ethnicity with Narrative Comments from Internal Medicine Resident Performance Assessments | | | | | | | |
| --- | --- | --- | --- | --- | --- | --- | --- |
|  | | Specificity of Comments | | | Valence of Comments | | |
|  |  | Beta Estimate† | Standard Error | P value | Beta Estimate† | Standard Error | P value |
| Resident Gender* | All years | -0.07 | 0.02 | 0.002 | 0.06 | 0.03 | 0.045 |
|  | PGY1 | -0.11 | 0.03 | <0.001 | 0.10 | 0.04 | 0.015 |
|  | PGY2 | -0.05 | 0.05 | 0.313 | 0.03 | 0.06 | 0.630 |
|  | PGY3 | 0.01 | 0.06 | 0.889 | 0.07 | 0.07 | 0.327 |
| * women residents compared to men residents  † Based on multilevel regression controlling for standardized composite competency rating, type of comment, comment characteristic (valence or specificity), program, resident characteristics (PGY, gender, race/ethnicity, and baseline In-Training Examination percentile rank), faculty characteristics (gender, department, and education role), rotation time | | | | | | | |
